# Sequence Permutation Generates Peptides with Different Antimicrobial and Antibiofilm Activities

**DOI:** 10.3390/ph13100271

**Published:** 2020-09-25

**Authors:** Biswajit Mishra, Jayaram Lakshmaiah Narayana, Tamara Lushnikova, Yingxia Zhang, Radha M. Golla, D. Zarena, Guangshun Wang

**Affiliations:** Department of Pathology and Microbiology, College of Medicine, University of Nebraska Medical Center, 985900 Nebraska Medical Center, Omaha, NE 68198-5900, USA; biswajit.dna@gmail.com (B.M.); jayaram.narayana@unmc.edu (J.L.N.); tlmustela@gmail.com (T.L.); yingxiazhang@hotmail.com (Y.Z.); radhamg@gmail.com (R.M.G.); zareenajntua@gmail.com (D.Z.)

**Keywords:** antimicrobial peptides, antibiotic resistance, database, peptide discovery, sequence permutation

## Abstract

Antibiotic resistance poses a threat to our society, and 10 million people could die by 2050. To design potent antimicrobials, we made use of the antimicrobial peptide database (APD). Using the database filtering technology, we identified a useful template and converted it into an effective peptide WW291 against methicillin-resistant *Staphylococcus aureus* (MRSA). Here, we compared the antibacterial activity and cytotoxicity of a family of peptides obtained from sequence permutation of WW291. The resulting eight WW peptides (WW291-WW298) gained different activities against a panel of bacteria. While WW295 inhibited the growth of *Escherichia coli*, WW298 was highly active against *S. aureus* USA300 LAC. Consistently with this, WW298 was more effective in permeating or depolarizing the *S. aureus* membranes, whereas WW295 potently permeated the *E. coli* membranes. In addition, WW298, but not WW295, inhibited the MRSA attachment and could disrupt its preformed biofilms more effectively than daptomycin. WW298 also protected wax moths *Galleria mellonella* from MRSA infection causing death. Thus, sequence permutation provides one useful avenue to generating antimicrobial peptides with varying activity spectra. Taken together with amino acid composition modulation, these methods may lead to narrow-spectrum peptides that are more promising to selectively eliminate invading pathogens without damaging commensal microbiota.

## 1. Introduction

Pathogenic microbes, ranging from viruses to bacteria, are life threatening. This can be seen from the current pandemic of COVID-19, which became a global crisis due to a lack of effective medicine. Likewise, antibiotic resistance can put patients in a dangerous situation when doctors have no access to effective drugs. It is clear that human beings face an unprecedented challenge from pathogenic microbes that cause a variety of infectious diseases. The situation worsens when these pathogens form biofilms, where numerous pathogens are encased in a biological matrix (polysaccharides, nucleic acids and proteins). Such biofilms are difficult to treat by conventional antibiotics. Consequently, effective antibiofilm agents are under active search [1].

Antimicrobial peptides (AMPs, also called host defense peptides or innate immune peptides) are promising candidates as they can eliminate antibiotic-resistant pathogens, including those in the biofilm form [2,3,4,5,6,7]. AMPs have been discovered in the three life domains: bacteria, archaea, and eukarya. They play a critical role in innate immune systems to protect plants, invertebrates and vertebrates from infection. In invertebrates such as insects, specific AMPs are induced depending on the type of invading pathogens. Here, innate immune peptides are critical because insects do not contain adaptive immune systems [8]. Currently, over 3000 natural innate immune peptides are registered in the Antimicrobial Peptide Database (APD) [9,10]. The majority of such peptides (88% in the APD) are small with less than 50 amino acids. On average, natural AMPs in the APD have a net charge of +3.3 and a hydrophobic content of 41.3%. Remarkably, these peptides could adopt a variety of structural scaffolds (α, β, αβ, and non-αβ) [11]. Based on the chain connection patterns, they are unified into four classes: (I) linear (e.g., LL-37), (II) sidechain-connected (e.g., defensins), (III) sidechain-backbone linked (e.g., daptomycin), and (IV) backbone-linked (e.g., circular cyclotides) peptides [11]. Due to sequence simplicity, linear peptides remain popular. Recently, we found length-dependent correlations with averaged peptide net charge or hydrophobic contents for over 1000 amphibian AMPs [12]. Many such linear peptides adopt a helical conformation in membrane-mimetic environments [13,14]. Because cationic helical peptides are usually amphipathic with two distinct surfaces, hydrophobic and cationic, they consist of a classic molecular design for targeting anionic bacteria. In contrast, mammalian cells are rich in zwitterionic phosphocholines, which are less attractive to cationic AMPs. Such a lipid composition difference between host and pathogen membranes is believed to be the molecular basis for the cell selectivity of cationic AMPs, a requirement as novel medicine [2,3,4,5,6,7].

The isolation of potent antimicrobials from natural sources is an important approach [2,3,4,5,6,7]. Although labor-intensive, this classic approach has the great potential to identify novel candidates, including peptides [15,16,17,18,19]. Recent efforts have extended the search of new antimicrobials to uncultivable bacteria and microbiota [18,19]. We have been utilizing database approaches, ranging from database screening to database filtering technology [20,21,22]. Recently, we found the importance of low cationicity for systemic efficacy of the database-designed peptides in mice against methicillin-resistant *Staphylococcus aureus* (MRSA) [23]. On the basis of this idea, we designed an even shorter peptide WW291 (formerly TetraF2W-RK) [24]. WW means tryptophan rich. This peptide forms an amphipathic helical structure in complex with lipid micelles [25]. Horine, an optimized peptide of WW291, indeed displays systemic efficacy in mice [26]. This study investigates the effects of sequence permutation on the cytotoxic and antimicrobial activities of WW291 using multiple bacteria and mammalian cells. Since WW295 and WW298 are more active against Gram-negative and Gram-positive bacteria, respectively, we compared the membrane permeation effects of these two peptides in this series against *E. coli* and MRSA. We also compared the antibiofilm capabilities of W295 and WW298 against MRSA. Finally, we showed in vivo efficacy of WW298 in protecting wax moths from bacterial infection caused by MRSA. This study suggests that sequence permutation offers a useful method to generate new antimicrobials against drug-resistant pathogens.

## 2. Results

### 2.1. In Silico Properties of Sequence-Permutated Peptides

With the development of bioinformatic tools, it becomes convenient to calculate peptide physical and chemical properties based on online computer programs. Using the ExPASy and APD websites, we calculated Boman index, GRAVY, aliphatic index, predicted half-life, and instability index [2,9,27] for the WW peptide family generated from sequence permutation [26], including WW291 to WW298 (Table 1). For averaged parameters that do not depend on peptide sequence, we obtained identical values for Boman index (originally called protein-binding potential by Boman [2]), GRAVY, and aliphatic index for all the peptides. Likewise, all these WW peptides share the same molecular formula C_68_H_88_N_16_O_9_, molecular weight 1273.55, molar extinction coefficient 22,000, and an isoelectric point (pI) 11. However, the predicted half-life and instability index of these peptides varied since such properties are related to peptide sequence, including the exposed N-terminal amino acid. These calculations predict that WW295 and WW296 have the shortest half-life in vivo (1–1.3 h), while WW297, with an isoleucine at the N-terminus, has the longest half-life of 20 h. This N-terminal end rule reveals that a peptide with an N-terminal valine would be most stable (half-life 100 h). In the case of instability index, it is defined that a peptide with an instability index less than 40 is stable. Based on these criteria, all the WW peptides in Table 1 are predicted to be stable, except for WW297, with an instability index of 48.25.

### 2.2. Antibacterial Activity

The antimicrobial activity spectrum of the WW peptide series was performed on a panel of Gram-positive and -negative bacteria using the standard broth micro-dilution method [28] with minor modifications as described [23]. The minimal inhibitory concentrations (MIC) of WW291-WW298 against eight bacteria are provided in Table 2. We may define the magnitude of antimicrobial activity change by taking the MIC ratio between the highest (less active) and the lowest (more active). There was a small change in the case of *Pseudomonas aeruginosa* and *Acinetobacter baumannii* up to two-fold. Four-fold changes were found for *Staphylococcus aureus*, *Staphylococcus epidermidis*, *Escherichia coli*, and *Bacillus subtilis*. However, we observed over eight-fold MIC changes for *Klebsiella pneumoniae* and vancomycin-resistant *enterococcus* (VRE). In both cases, WW295 appeared to be more active (Table 2). It is also slightly more active against *E. coli*. In contrast to other Gram-negative bacteria, these WW peptides displayed poor antimicrobial activity against *P. aeruginosa* (MIC 25–50 µM). We also observed more than eight-fold changes in minimal bacterial killing concentrations. WW298 was most potent against Gram-positive MRSA USA300 in terms of minimal bactericidal concentration (MBC) (Table 2). These results suggest that WW295 is an antibacterial peptide against Gram-negative pathogens, while WW298 is best to combat MRSA.

To gain additional insight, we also compared the effects of physiological saline and human serum on the antibacterial activities of WW291-WW298 using both *E. coli* and *S. aureus* (Table 3). In our assays, salts and serum were present during the entire course of the experiment. It appeared that 150 mM NaCl had a relatively small influence on MIC values, especially when *S. aureus* USA300 LAC was utilized. Human serum had a clear impact on the antimicrobial activity of these peptides. However, WW295 retained a good antibacterial activity (MIC 12.5 µM) against both *E. coli* and *S. aureus* in 10% serum, confirming its potential as a novel antimicrobial template. Of note, WW296 and WW298 retained some antibacterial activity against MRSA in 10% serum (MIC 12.5–25 µM). Thus, WW295 and WW298 stand out in terms of activity robustness (bold in Table 3).

### 2.3. Cytotoxicity Assessment

**Figure 1:**
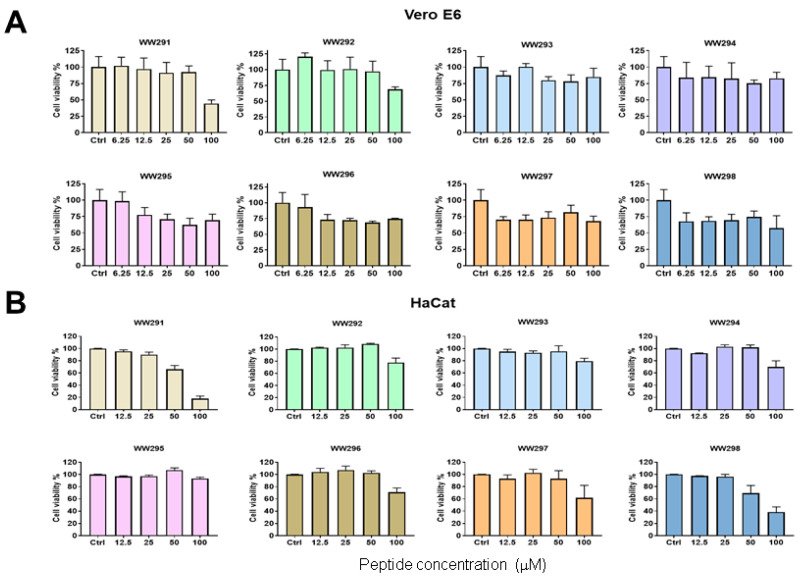
Assessment of the cytotoxicity of sequence permutated WW peptides on monkey kidney Vero E6 cells (**A**) and human epidermal keratinocyte cell line (HaCaT) (**B**). Peptides were tested at different concentrations (6.25 to 100 µM in panel A and 12.5−100 µM in panel (**B**)). Cell viability was quantitated using the standard MTS assay as described in Methods.

**Figure 2:**
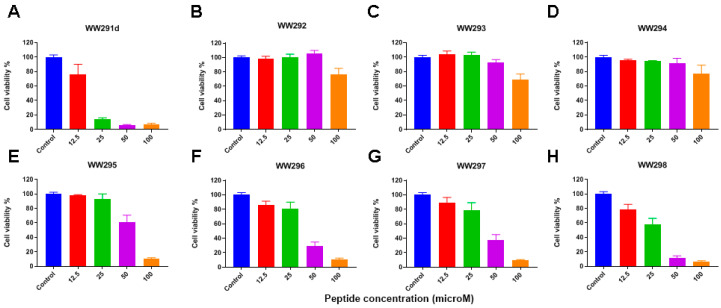
Assessment of the cytotoxicity of sequence-permutated peptides WW291-298 on human monocytes (THP-1). Panels (**A**–**H**) represent cell viability after each peptide treatment at concentrations in the range of 12.5 to 100 µM. Cell viability was quantitated using the standard MTS assay (see Methods).

In order to achieve an understanding of the cytotoxic behavior of the WW peptide series, we tested their effects on numerous host cells (Table 4). These include human, chicken, bovine red blood cells (RBC), monkey kidney Vero cells (Figure 1A), human keratinocytes (HaCaT) (Figure 1B), and PMA differentiated THP-1 cells (Figure 2). WW292–WW294 were less hemolytic than other peptides, especially WW291, WW295, and WW298 (Table 4). In the case of Vero cells, WW291 and WW292 showed little toxicity below 100 µM, while other peptides caused 25% cell death at 6.2–12.5 µM. These peptides were slightly less toxic to skin cells, with essentially no cell death below 25–50 µM. However, human monocyte-derived macrophages (dTHP-1) were more susceptible to WW291 (D-form made of D-amino acids), WW295–WW298, in a dose-dependent manner. We estimated the peptide concentration that caused 50% cell lysis (HC_50_) or 50% cell death (LC_50_) and the results are summarized in Table 4. Notably, the cytotoxicity profiles of the peptides were found to be analogous to the microbicide activity. Peptides WW292–WW294 less active against bacteria were also less toxic to mammalian cells, since the LC_50_ and HC_50_ were found to be greater than 100 µM. More potent antibacterial peptides, like WW295 and WW298, retained more cytotoxic character, like the parent peptide WW291 with LC_50_ values less than 100 µM to some mammalian cell lines. Of outstanding interest is that all these peptides displayed poor toxicity to kidney Vero cells (Figure 1A) with LC_50_ equal to 100 µM or greater (Table 4). Because WW295 and WW298 are more potent against *E. coli* and *S. aureus*, respectively (Table 2), with robust activity in the presence of salts and human serum (Table 3), and their HC_50_ to dTHP-1 (more susceptible) are ~8-fold higher, they were chosen for further characterization.

### 2.4. Propidium Iodide-Based Membrane Penetration Assay

**Figure 3:**
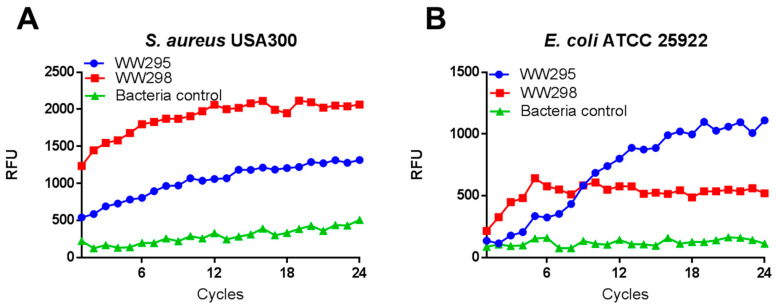
Membrane permeation assay of WW295 and WW298 against (**A**) *S. aureus* USA300 and (**B**) *E. coli*. The plots show the fluorescence buildup of propidium iodide with time due to the treatment of the peptide at 3.1 µM. Each cycle is 5 min, leading to a total of 2 h. Bacteria cells without any treatment were used as controls to compare effective permeation.

Although not general, cationic antimicrobial peptides frequently target bacterial membranes [2,3,4,5,6,7]. A fluorescence-based assay was performed for the determination the membrane active nature of peptides. A rapid increase in the fluorescence was detected as a consequence of peptide treatment at 3.1 µM, indicating membrane damage (Figure 3) [23]. In the case of *S. aureus* USA300 (Figure 3A), the peptide WW298 showed a time-dependent fluorescence buildup, much higher than that of WW295. For *E. coli*, a steady fluorescence buildup of WW295 was prominent (Figure 3B). These results are in line with the antibacterial activity that WW298 is more active against MRSA than WW295 (Table 2).

### 2.5. Simultaneous Detection of the Outer and Inner Membrane Permeation of E. coli

**Figure 4:**
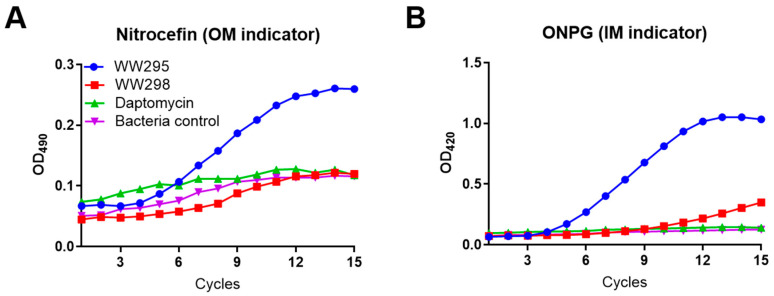
Simultaneous permeation of the outer and inner membranes in the *E. coli* ML35p reporter strain by peptide WW295 and WW298. The detection of nitrocefin at 490 nm as a product of beta-lactamase cleavage is a reporter for the outer membrane (**A**) and cleavage of ONPG by the cytoplasmic β-galactosidase into o-nitrophenol, monitored at 420 nm is the evidence for the inner membrane permeation (**B**). Plots shown here are at 3.1 µM of peptide concentration plotted as a function of relative fluorescence units with time. Each cycle is of 5 min. Bacteria cells without any treatment were used as controls.

To confirm the mechanism of action, we also performed membrane permeation using a reporter strain of *E. coli* ML35p [29]. This strain enabled simultaneous monitoring of membrane permeation of both the inner and outer membranes of Gram-negative bacterium *E. coli*. Both WW295 and WW298 were included in the experiment. At a sub-micromolar concentration of 3.1 µM, the peptide WW295 entered both outer and inner membranes (Figure 4). This is indicated by the sharp development of absorbance at 490 nm for nitrocefin (panel A) and at 420 nm for ONPG (panel B). In the same experiment, both WW298 and daptomycin were poor in membrane permeation, similar to that of untreated bacteria. This is consistent with activity data where WW295 is more active against *E. coli* than WW298 (Table 2), while daptomycin is primarily active against Gram-positive bacteria.

### 2.6. Evidence of Membrane Depolarization of S. aureus USA300

**Figure 5:**
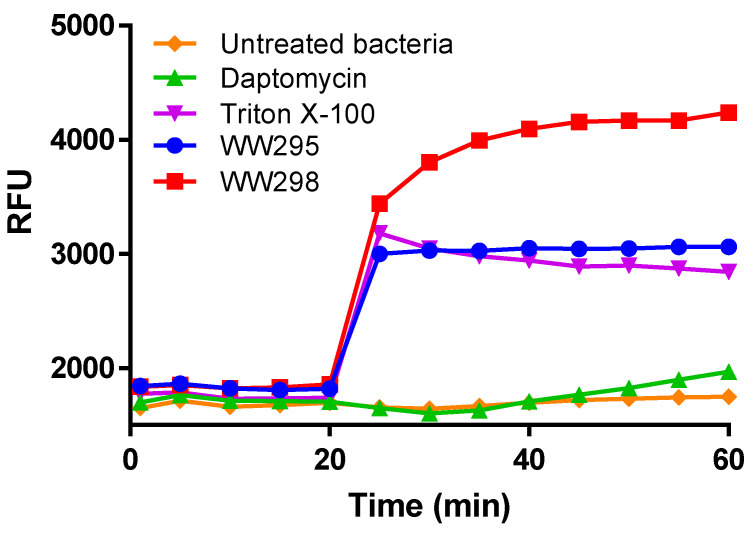
Membrane depolarization of *S. aureus* USA300 by WW295 and WW298. Plots shown here are at 12.5 µM of peptide concentration plotted as a function of relative fluorescence units with time.

Bacteria surfaces are negative and cationic peptides are able to neutralize the negative charge, thereby reducing membrane potential. To test this possibility, we evaluated the peptides against *S. aureus* USA300 LAC using a fluorescent indicator dye, the DiBAC_4_(3) (Figure 5). A rapid increase in fluorescence upon the action of peptides on energetic bacterial cells is a direct indication of the bacteria surface charge neutralization as a measure of shift in the electric charge distribution. Observed at 12.5 µM, the peptide WW298 had the highest fluorescence development whereas WW295 was less active in depolarizing the membranes of *S. aureus*, but comparable to the positive control Triton-X100. In contrast, daptomycin at this concentration has significantly less membrane depolarization under the same experimental conditions.

### 2.7. Antibiofilm Effects on MRSA

**Figure 6:**
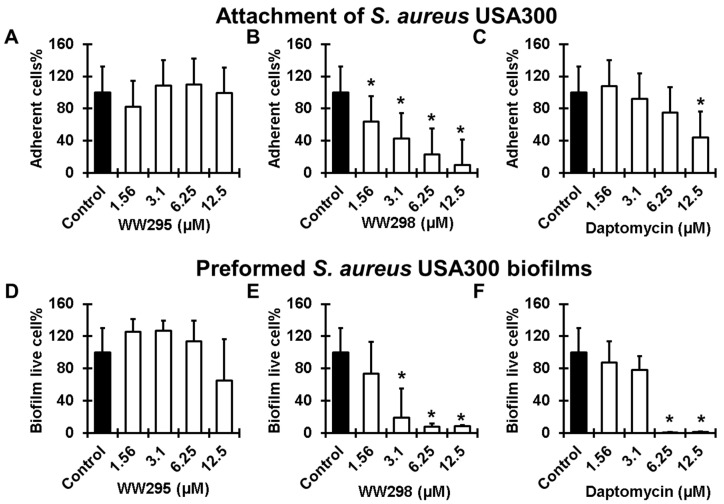
Concentration-dependent antibiofilm capability of WW295 and WW298 against *S. aureus* USA300 LAC in comparison to daptomycin. Inhibition of the first stage of attachment (adherence) of high-density bacteria onto polypropylene substrates (**A**–**C**), and disruption of 24 h matured biofilms (**D**–**F**) by WW295, WW298 and daptomycin, respectively. Statistical significance was calculated using a paired student *t*-test, and a *p*-value < 0.05 was considered significant (*).

Antimicrobial effects of the peptides are different on diverse forms of bacteria. The free and motile forms cultivated in laboratories are the planktonic bacteria onto which the peptides and antibiotics can have direct effects. However, the activity of the microbicides can be reduced to the biofilm forms where bacteria are less exposed and hidden inside the shield. The development of the biofilms starts with the attachment of the individual cells on the substratum followed by biofilm growth and maturation into more solid biomass. We tested the ability of WW295 and WW298 in fighting the biofilms of *S. aureus* USA300 LAC at the two stages. While the peptide WW295 did not show any effects (Figure 6A), WW298 was found to be more active (Figure 6B) than the currently used peptide antibiotic daptomycin in inhibiting the surface adherence of MRSA on polypropylene plates (Figure 6C). These 96-well commercial plates were pre-treated commercially with tissue culture to enhance cell adhesion. Of note, WW298 was found to be active at sub-MIC concentration of 1.56 µM where it inhibited ~40% bacterial adherence. At 3.1, 6.25 and 12.5 µM, it inhibited ~50, 80, and 90% MRSA adherence, respectively. In contrast, daptomycin was less effective and only inhibited ~50% cell adherence at 12.5 µM.

**Figure 7:**
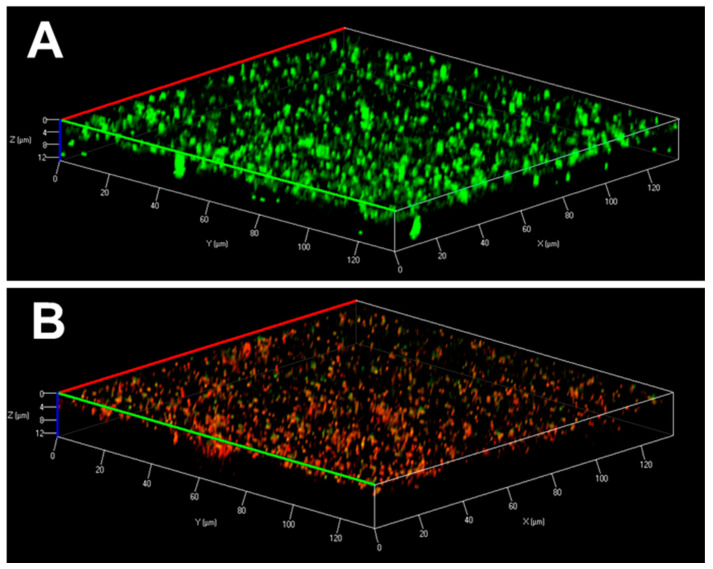
Confocal microscopic images of the 24 h established biofilms of *S. aureus* USA300 LAC without (**A**) and with peptide treatment (**B**). Biofilms were stained using the live and dead staining kit containing a mixture of 4′,6-diamidino-2-phenylindole (DAPI, green) and propidium iodide (PI, red). Both dyes bind bacterial DNA, a molecular process that only occurs when bacterial membranes are damaged by the peptide. Bacteria in the untreated control biofilms are green and live (**A**), whereas the bacteria are dead and turned red (**B**) after treatment with WW298 at 12.5 µM.

We also compared the effects of the peptides on established biofilms (24 h). While WW295 did not disrupt MRSA biofilms significantly even at the highest concentration (12.5 µM) we tested (Figure 6D), WW298 disrupted ~80% at 3.1 µM and more than 95% of established biofilms at 6.25 µM (Figure 6E). As a positive control, daptomycin, a bacteria-derived peptide antibiotic, was effective in eradicating almost all the established biofilm at 6.25 µM, although it was much less effective than WW298 at 3.1 µM (Figure 6F). Confocal microscopy revealed the presence of the dead cells in the peptide treated biofilms (at 12.5 µM) after staining with a live and dead kit (Figure 7). When cells are alive (in the absence of peptide), the cell-permeant calcein-AM dye could enter the cells and gave green color as a consequence of the removal of acetoxymethyl (AM) esters by intracellular esterase (Figure 7A). When the cells were dead due to membrane damage by the peptide, the red dye ethidium homodimer (non-membrane permeable) could enter the cell and become red by binding to nucleic acids (Figure 7B).

### 2.8. Protection of Galleria Mellonella Wax Moths from MRSA Infection

**Figure 8:**
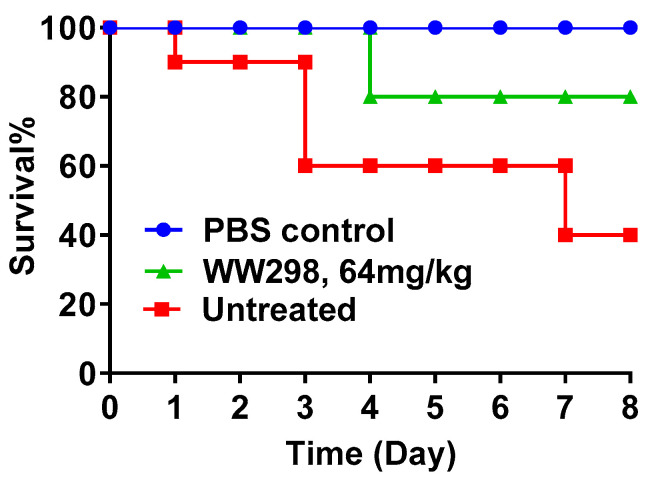
Protection of *Galleria mellonella* (wax moth) from *S. aureus* USA300 infection by WW298. The animals were infected 2 h prior to treatment with PBS or peptide. PBS was injected into a control set of worms to ensure peptide treatment being the only cause of worm survival upon infection.

We then moved onto testing the in vivo efficacy of WW298 since it is potent against MRSA. For this purpose, we used the wax moth infection model [30,31]. To observe the protective effect of the peptide, WW298 was injected into the wax worms two hours prior to infection with *S. aureus* USA300 LAC (1 × 10^6^ CFU/moth). In the entire experiment, we did not observe any moth death in the uninfected control group treated with PBS for 8 days. In the infected group, 60% of the insects died by day 8. In contrast, only 20% died and 80% survived in the peptide treated group (64 mg/kg). Hence, WW298 treatment protected the majority of wax moths from death (Figure 8).

## 3. Discussion

Amino acid sequence determines protein structure and function. Sequence scrambling is a standard practice to destroy protein function. This appears to work well when a specific polypeptide sequence is required to recognize a protein partner. In the case of antimicrobial peptides, sequence scrambling may not destroy peptide antimicrobial activity completely, especially when they target bacterial membranes. This is because the chiral requirement for membrane targeting is less strict. A classic experiment to prove this is to synthesize a mirror image of AMPs using D-amino acids. The two versions of the same peptide kill bacteria equally well when the target is membrane [2]. This is indeed the case for the two membrane-targeting peptides, horine and verine, based on the MIC values of the L- and D-forms [26]. In contrast, very different activities would suggest a non-membrane target. It is known that even sequence reversal can make a difference. While the sequence-reversed LL-37 core peptide is active against bacteria, it loses activity against human immunodeficiency virus type 1 (HIV-1) [32,33]. Nevertheless, sequence shuffling (i.e., in silico evolution) is useful to generate new AMPs [21,34,35,36,37]. We found previously that sequence shuffling alters peptide anti-HIV activity. Since all three possibilities were observed: activity increase, similar activity, and activity decrease, sequence shuffling presents one useful avenue to peptide activity improvement [21].

Different from sequence shuffling, which can cause a drastic recombination of amino acids in a peptide sequence, sequence permutation only introduces a mild change in each operation. To carry this out, one can first connect the N- and C-termini of the peptide so that the peptide becomes circular. Then, a break is introduced into the circle, one at a time, until all the peptide bonds have a chance to be cut. Another way to practice this is to move one amino acid from the N-terminus to the C-terminus, one at a time, until the same sequence recurs [26]. As a consequence, sequence permutation only produces a limited number of new peptides depending on the peptide length. The relationships between the amino acids are also largely maintained. However, this practice does generate novel sequence patterns [26]. Our sequence permutation of WW291, a small peptide with eight amino acids, generated eight peptides. As shown in Table 1, sequence permutation may alter peptide physical and chemical properties. The predicted peptide half-life changed due to the appearance of different residues at the N-terminus. Likewise, permutation modulated instability index due to the variation in the type of dipeptides in the sequence. However, Boman index, GRAVY, and aliphatic index remained the same because these averaged values depend on peptide amino acid composition rather than peptide sequence. Sequence permutation also influenced the physical interaction of each peptide with the hydrophobic phase of the reverse-phase HPLC column, leading to slight differences in retention times (Table 1). The longer retention times for WW294 and WW295 implied slightly higher hydrophobicity [38,39,40].

Sequence permutation also altered biological activities of the peptides. Interestingly, the magnitude of variation is pathogen-dependent (Table 2). We found recently that sequence permutation could transform the peptide three-dimensional structure, leading to distinct amphipathic structures, providing a basis for the change of peptide activity spectrum [26]. Hence, this practice may open the door to identification of peptides with a desired activity spectrum against targeted pathogens. Such a fine-tuning of antimicrobial activity spectrum is currently not fashionable, but is important for developing future precise antibiotics that selectively eliminate the pathogens of concern, thereby preserving the microbiota to our benefit. For instance, horine, a refined peptide of WW291 with activity mainly against Gram-positive bacterial species [26], may be used to selectively eliminate MRSA and VRE when they become offensive pathogens in patients. Our previous database discovered anti-MRSA peptide DFTamP1 [22], as well as its optimized peptides DFT503 and DFT561 [23], may serve the same purpose. In the current APD [9], 551 AMPs are annotated to be primarily active against Gram-positive bacteria. These include some bacteriocins synthesized and secreted by Gram-positive bacteria to inhibit competitive strains [16,17,18,19]. In contrast, 316 AMPs in the APD are reported to be active primarily against Gram-negative pathogens [9]. In addition to sequence permutation, peptide activity spectrum can also be altered by tuning amino acid composition [31]. We found it possible to convert the major antimicrobial peptide of human cathelicidin LL-37 into a selective peptide GF-17d3, active mainly against Gram-negative *E. coli* and *A. baumannii* [38,41]. There are also alternative methods to eliminate pathogens selectively. Commensal bacteria in human microbiota can stop the invading pathogens [19]. Bacteriophages can target specific bacterial strains, including drug-resistant pathogens [42].

## 4. Materials and Methods

### 4.1. Peptides and Property Calculations

All peptides were chemically synthesized and purified to >95% (Genemed Synthesis, San Antonio, TX, USA). The quality of each peptide is indicated by Mass Spectroscopy and HPLC. Fresh stock solutions were made by solubilizing peptides in autoclaved distilled water and the concentrations of tryptophan-containing peptides were determined by UV spectroscopy at 280 nm [43]. Other chemicals were purchased from Sigma (St. Louis, MO, USA) unless specified. Peptide Boman index [2], GRAVY [44], aliphatic index [45], predicted half-life [46], and instability index [47] were calculated using the antimicrobial peptide database prediction interface (*http://aps.unmc.edu/AP/prediction/prediction_main.php*) [9,48] and ExPASy (*https://web.expasy.org/protparam/*) [27].

### 4.2. Bacterial Strains and Growth Media

The bacterial strains used in this study included the Gram-positive strains *Staphylococcus aureus* USA300 LAC, *S. aureus* Mu50, *S. epidermidis* 1457, *B. subtilis* 167, *E. faecium* VRE as well as the Gram-negative isolates *Escherichia coli* ATCC 25922, *E. coli* K12, *Pseudomonas aeruginosa* PAO1, *A. baumannii* B28-16, and *Klebsiella pneumoniae* ATCC 13883.

### 4.3. HPLC Retention Time Measurements

The retention time of the peptide was measured on a Waters HPLC system equipped with an analytical reverse-phase WATERS C_18_ column (4.6 × 250 mm). The peptide detected at 220 and 280 nm was eluted with a gradient of acetonitrile (containing 1% TFA) from 5% to 95% at a flow rate of 1 mL/min [22].

### 4.4. Antimicrobial Assays

The antibacterial activity of peptides was evaluated using a standard broth microdilution protocol with minor modifications as previously described [23]. In brief, logarithmic phase bacterial cultures (i.e., optical density at 600 nm ≈ 0.5) were diluted to OD_600_~0.001 and partitioned into a 96-well polystyrene microplate at 90 μL per well. After treatment with 10 μL of peptide solutions two-fold diluted to various concentrations, microplates were incubated at 37 °C overnight and read on a ChroMate 4300 Microplate Reader at 630 nm (GMI, Ramsey, MN). The minimal inhibitory concentration (MIC) is the lowest peptide concentration that fully inhibited bacterial growth. Minimal bactericidal concentration (MBC) was determined by plating the clear wells at MIC or above. The peptide concentration that caused no bacterial growth on the petri dish is MBC.

### 4.5. Hemolytic Assays

Hemolytic assays of peptides were performed as previously described [23]. Briefly, human red blood cells (hRBCs) obtained from UNMC Blood Bank, or chicken or porcine blood cells purchased from commercial sources as previously described [24], were washed three times with phosphate buffer saline (PBS) and diluted to a 2% solution (V/v). After peptide treatment, incubation at 37 °C for one hour, and centrifugation at 13,000 rpm, aliquots of the supernatant were carefully transferred to a fresh 96-well microplate. The amount of hemoglobin released was measured at 545 nm. The percent lysis was calculated by assuming 100% release when human blood cells were treated with 2% Triton X-100, and 0% release when incubated with PBS buffer. The peptide concentration that caused 50% lysis of hRBCs is defined as HC_50_.

### 4.6. Toxicity of Peptides on Mammalian Cells

Peptides were assayed for potential in vitro toxicity against human keratinocytes (HaCaT) and THP-1 cells as previously described [24]. THP-1 monocytes were differentiated into macrophages by adding 30 ng/mL of phorbol 12-myristate 13-acetate (PMA; Sigma-Aldrich) to RPMI 1640 supplemented with 10% fetal calf serum culture (FCS) medium for 16 h. Briefly, cells were seeded at a density of 3 × 10^4^ per well in a tissue culture 96-well plate in DMEM/RPMI supplemented with 10% fetal bovine serum (FBS), and incubated at 37 °C in a 5% CO_2_ atmosphere for 24 h. African green monkey kidney Vero E6 cells were cultivated in EMEM in the same manner. Before antimicrobial treatment, wells were replaced with serum free media and treated with peptides at different concentrations for one hour. After incubation, the cells were washed and incubated with 100 μL of DMEM media containing 20 μL of 3-(4,5-dimethylthiazol-2-yl)-5-(3-carboxymethoxyphenyl)-2-(4-sulfophenyl)-2*H*-tetrazolium (MTS) reagent for two hours at 37 °C. The treatment time was one hour in the case of Vero E6 cells. Absorbance readings at 492 nm were taken using a ChroMate microplate reader (GMI, Ramsey, MN, USA).

### 4.7. Real-Time Fluorescence-Based Kinetics of Bacterial Killing

The experiment was performed as previously described with minor modifications [26]. Serially diluted 10× peptides (10 μL each well) were created in 96-well microtiter plates. Propidium iodide (2 μL) at a fixed concentration of 20 μM was added to each well followed by 88 μL of the *S. aureus* USA300 or *E. coli* ATCC 25922 culture (a final OD600 ~0.1 in PBS). The plate was incubated at 37 °C with continuous shaking at 100 rpm in a FLUOstar Omega (BMG LABTECH Inc, Cary, NC, USA) microplate reader. The fluorescence from the plate was read every 5 min for a total duration of 2 h with excitation and emission wavelengths of 584 and 620 nm, respectively. Data were analyzed using the MARS data analysis software provided by the manufacturer and representative plots were made using the GraphPad Prism software (version: 7.0).

### 4.8. Simultaneous Penetration of the Bacterial Outer and Inner Bacterial Membranes

The *E. coli* ML-35p strain was utilized to probe the permeation of both the outer membrane (OM) and inner membrane (IM) [29]. This strain was engineered to have two membrane reporters. Nitrocefin is used for detection of OM permeabilization. Nitrocefin is excluded from the periplasm. However, it enters the periplasmic space and gets cleaved by β-lactamase when bacterial OM is permeabilized by innate immune peptides. The chromogenic cleavage product was spectrophotometrically monitored at 490 nm. For IM permeation, o-nitrophenylgalactose (ONPG) is used as a reporter. ONPG is debarred to enter cytoplasm due to the lack of the lac permease in this strain. However, permeabilization of the IM helps ONPG to enter cytoplasm and get cleaved by cytoplasmic β-galactosidase into o-nitrophenol, which can be detected spectrophotometrically at 420 nm. Experimentally, bacteria from an overnight culture, after re-inoculated into fresh TSB, were grown to the exponential phase at 37 °C for 2 h with rotation at 220 rpm. After washing with 1 × PBS (GIBCO, Life Technologies Corporation, NY, USA), the culture was diluted to ~10^8^ CFU mL/mL in PBS with 1% TSB. 70 µL of this culture was added to a 96-well microtiter plate (Corning Costar, Amsterdam, The Netherlands) containing increasing concentrations of peptide, 30 × 10^−6^ M nitrocefin and 2.5 × 10^−3^ M ONPG. Absorbances at both 490 and at 420 nm were monitored using FLUOstar Omega (BMG, Labtech, NC, USA) simultaneously. After shaking at 100 rpm, readings were taken every 2 min for a total of 40 min at 37 °C. Data were analyzed using the MARS data analysis software provided by the manufacturer and representative plots were made using the GraphPad Prism 7 software.

### 4.9. Membrane Depolarization of Bacteria

The overnight culture of *S. aureus* USA300 LAC was grown in TSB media to the exponential phase. Cells were then pelleted and washed with PBS twice, and re-suspended in twice the volume of PBS containing 25 mM glucose for 15 min at 37 °C. For membrane depolarization measurements, 250 nM (final concentration) of the dye DiBAC_4_(3) (bis-(1,3-dibutylbarbituric acid) trimethine oxonol (ANASPEC, Fremont, CA, USA) was added, and vortexed gently. Ninety microliters of the energized bacteria solution were then added in a 96-well microtiter plate (Corning COSTAR). The plate was immediately placed into a FLUOstar Omega (BMG LABTECH Inc., Cary, NC, USA) microplate reader and fluorescence was measured for 20 min. Once the readings are stabilized, 10 μL of peptide solution were added and the fluorescence readings were continued for another 40 min. Measurements were done at an excitation and emission wavelengths of 485 and 520 nm, respectively. Triton X-100 (0.1%) was included as a positive control [49].

### 4.10. Peptide Effects on Biofilm Attachment

This experiment was performed to measure the ability of the peptide to inhibit bacterial attachment, the initial step for biofilm formation. In short, an overnight culture of *S. aureus* USA300 was grown in TSB media to an optical density (600 nm) of ∼1.0. 180 μL of this culture were added to each well of the microtiter plates containing 20 μL of 10× peptide solution. The plates were then incubated at 37 °C for 1 h. Media with bacteria were then pipetted out and chambers were washed with 1× PBS to remove non-adherent cells. Live cells in the biofilms were then quantitated using XTT [2,3-bis(2-methyloxy-4-nitro-5-sulfophenyl)-2*H*-tertazolium-5-carboxanilide] assay by following the manufacturer’s instructions with modifications. A total of 180 μL of fresh TSB and 20 μL of XTT solution were added to each well and the plates were again incubated at 37 °C for 2 h. Absorbance at 450 nm (only media with XTT containing wells served as the blank) was obtained using a Chromate^TM^ microtiter plate reader. Percentage biofilm growth for the peptide was plotted by assuming 100% biofilm growth is achieved on the bacterial wells without peptide treatment.

### 4.11. Antibiofilm Effects on 24 h Established Bacterial Biofilms

The antibiofilm activity of the peptide against 24 h established biofilms was evaluated as previously described [24]. In short, *S. aureus* USA300 LAC (10^5^ CFU/mL) was made from exponential phase bacteria in TSB media and 180 μL were distributed to each well of the microtiter plate (Corning Costar Cat No. 3595, Amsterdam, The Netherlands). The plates were incubated at 37 °C for 24 h to form a biofilm. Culture treated with water served as a positive control while media without bacterial inoculation served as the negative control. Media were then pipetted out and the attached biofilms were washed with 1× PBS to remove the planktonic bacteria. Each well of the plate was then filled with 20 μL of 10× peptide solution and 180 μL of fresh TSB media and the plates were further incubated at 37 °C for 24 h. Media were then pipetted out and the wells were washed with 1× PBS to remove planktonic cells. The live cells in biofilms were estimated by XTT assay as mentioned above.

### 4.12. Confocal Microscopy Observation of Live and Dead Bacteria in Established Biofilms after Peptide Treatment

*S. aureus* USA300 LAC (10^5^ CFU/mL) was made from the exponentially growing bacteria. To the chambers of cuvette, 1800 μL were added (Borosilicate cover glass systems, Nunc Cat. No: 155380, Rochester, NY, USA) and were incubated for 24 h at 37 °C for biofilm formation. Media were then pipetted out and chambers were washed with 1× PBS to remove non-adhered cells. To test the peptide effect on the preformed biofilms, 200 μL of 10× stocks of the peptide were added followed by 1800 μL of TSB. Control cuvettes were treated with water instead of peptide. The cuvettes were again incubated for another 24 h at 37 °C. The next day the supernatant was pipetted out and the chambers were washed with 1#xD7; PBS. The biofilms were stained with 10 μL of the LIVE/DEAD kit (Invitrogen Molecular Probes, Waltham, Massachusetts, MA USA) according to the manufacturer’s instructions. The samples were examined with a confocal laser scanning microscope (Zeiss 710) and the data were processed using Zen software (version: 2010).

### 4.13. Protection of Invertebrates from Death

The potency of WW298 was tested in vivo using an established wax moth *Galleria mellonella* model [30,31]. The animals (~250 mg) were distributed by Timberline Live Pet Foods Marion, IL. Peptide in phosphate buffer (64 mg/kg) was injected 2 h prior to the infection with *S. aureus* USA300 at 1 × 10^6^ CFU/moth. The animal groups included (1) PBS treated, (2) *S. aureus* USA300 infected without treatment, and (3) peptide treated prior to *S. aureus* infection. Animals (10 per group) were kept at room temperature and observed daily for 8 days to record live and dead ones.

### 4.14. Statistics

All experiments were replicated at least twice. For hemolysis, cytotoxicity and biofilms, plots represent the average values with standard deviation error bars. Membrane permeation experiments were duplicated for each condition and processed using the vendor’s software (MARS, BMG Labtech). For all the experiments, the level of significance was determined by performing paired Student’s *t*-test with parameters of two-tailed distribution (*p*-values > 0.05 were considered significant *).

## 5. Conclusions

In closing, there are two basic strategies to modulate peptide activity spectrum. The first is to alter peptide amino acid composition (i.e., charge or hydrophobicity). Using peptides derived from the major antimicrobial region of human cathelicidin LL-37, we previously demonstrated that a decrease in basic charge led to reduced antimicrobial activity against Gram-negative pathogens such as *A. baumannii*, whereas a decrease in hydrophobic amino acids under the same charged molecular frame reduced peptide activity against Gram-positive pathogens such as MRSA [31]. This study illustrates a second approach via changing peptide amino acid sequence (e.g., sequence permutation). At a constant peptide composition, WW298 is rather potent against MRSA, while WW295 gains activity against Gram-negative bacteria *E. coli*. We propose that the narrow-spectrum peptides generated by these approaches are in a better position as future precise medicine than wide-spectrum peptides in selectively eliminating the pathogens of concern with a minimal damage to human microbiota.

## Figures and Tables

**Table 1:** Amino acid sequences, calculated properties and measured HPLC retention times of sequence permutated peptides WW291-WW298.

Peptide	Sequence	Boman Index ^a^	GRAVY ^a,b^	Aliphatic Index ^b^	Half-Life ^b^	Instability Index ^b^	HPLC (t_r_, min) ^c^
WW291	WWWLRKIW	0.16	−0.46	97.5	2.8 h	37.64	ND ^d^
WW292	WWLRKIWW	0.16	−0.46	97.5	2.8 h	37.64	11.849
WW293	WLRKIWWW	0.16	−0.46	97.5	2.8 h	37.64	11.921
WW294	LRKIWWWW	0.16	−0.46	97.5	5.5 h	22.21	12.202
WW295	RKIWWWWL	0.16	−0.46	97.5	1.0 h	13.56	12.453
WW296	KIWWWWLR	0.16	−0.46	97.5	1.3 h	37.64	11.941
WW297	IWWWWLRK	0.16	−0.46	97.5	20 h	48.25	11.652
WW298	WWWWLRKI	0.16	−0.46	97.5	2.8 h	37.64	11.892

^a^ Calculated using the APD tool (*http://aps.unmc.edu/AP/prediction/prediction_main.php*); ^b^ Calculated from ExPASy (*https://web.expasy.org/protparam/*). Half-life was estimated based on mammalian reticulocytes in vitro. ^c^ t_r_ is the HPLC retention time of the peptide; ^d^ ND, not determined.

**Table 2:** Antibacterial activities of the WW peptides against various bacteria.

Peptide	Minimal Inhibitory Concentration (MIC, μM)								MBC (μM)
	SA	SE	BS	VRE	EC	PA	KP ^a^	AB	USA300
WW291	3.1	3.1–6.25	6.25	25	6.2–12.5	50	12.5	6.25	6.2
WW292	12.5	6.2–12.5	12.5	>50	25	25	>50	12.5	>25
WW293	12.5	6.2	12.5	50	12.5–25	50	25	12.5	25
WW294	12.5	6.2	25	25	12.5	50	6.2–12.5	12.5	25
WW295	12.5	6.2	12.5	6.25	6.2	25–50	3.1–6.2	6.25–12.5	12.5
WW296	3.1	3.1	12.5	25	6.2–12.5	50	12.5	12.5	6.2
WW297	6.2	3.1	12.5	12.5	12.5–25	50	12.5–25	6.25–12.5	12.5
WW298	3.1	1.5–3.1	6.25–12.5	12.5	12.5	50	6.2–12.5	6.25–12.5	3.1

^a^ Data for KP were taken from [19]. SA, *S. aureus* Mu50; SE, *S. epidermidis* 1457; BS, B. subtilis 167; VRE, E. faecium VRE; EC, *E. coli* K12; PA, *P. aeruginosa* PAO1; KP, *K. pneumonia* ATCC 13883; AB, *A. baumannii* B28-16; USA300, *S. aureus* USA300; MBC, minimal bactericidal concentration.

**Table 3:** Effects of salt and serum on peptide activity (in µM).

Peptide	TSB	+150 mM NaCl	+10% Human Serum
*E. coli* ATCC 25922			
WW291	**6.2–12.5**	**12.5**	>25
WW292	12.5	>25	>25
WW293	12.5	>25	>25
WW294	12.5	25	>25
WW295	**3.1**	**6.2**	**12.5**
WW296	3.1–6.2	25	>25
WW297	12.5	12.5	>25
WW298	**12.5**	**12.5**	>25
*S. aureus* USA300 LAC			
WW291	3.1	3.1	25
WW292	6.2	6.2	>25
WW293	6.2	6.2	>25
WW294	6.2	6.2	25
WW295	**3.1**	**3.1**	**12.5**
WW296	1.6	1.6	12.5
WW297	3.1	3.1	25
WW298	**1.6**	**1.6**	**12.5–25**

**Table 4:** Toxicity of the WW peptides to red blood cells or mammalian cells ^a^.

Peptide	Human RBC (µM)	Chicken RBC (µM)	Pig RBC (µM)	HaCaT (µM)	THP-1 (µM)	Vero Cells (µM)
WW291	30	50	ND	60	<25 ^a^	100
WW292	175	>100	>100	>100	>100	>100
WW293	224	>100	>100	>100	>100	>100
WW294	143	>100	>100	>100	>100	>100
WW295	57	100	>100	80	50	>100
WW296	103	50	>100	100	35	>100
WW297	134	100	110	100	38	>100
WW298	52	70	37	80	30	100

^a^ Concentrations in micromoles that caused 50% red blood cell (RBC) lysis or cell death.
